# A convolutional neural network for fully automated blood SUV determination to facilitate SUR computation in oncological FDG-PET

**DOI:** 10.1007/s00259-020-04991-9

**Published:** 2020-10-01

**Authors:** Pavel Nikulin, Frank Hofheinz, Jens Maus, Yimin Li, Rebecca Bütof, Catharina Lange, Christian Furth, Sebastian Zschaeck, Michael C. Kreissl, Jörg Kotzerke, Jörg van den Hoff

**Affiliations:** 1 Helmholtz-Zentrum Dresden-Rossendorf, PET Center, Institute of Radiopharmaceutical Cancer Research, Bautzner Landstrasse 400, 01328 Dresden, Germany; 2 Department of Radiation Oncology, Xiamen Cancer Center, The First Affiliated Hospital of Xiamen University, Xiamen, China; 3 OncoRay – National Center for Radiation Research in Oncology, Faculty of Medicine and University Hospital Carl Gustav Carus, Technische Universität Dresden, Helmholtz-Zentrum Dresden-Rossendorf, Dresden, Germany; 4 Department of Radiotherapy and Radiation Oncology, Faculty of Medicine and University Hospital Carl Gustav Carus, Technische Universität Dresden, Dresden, Germany; 5 National Center for Tumor Diseases (NCT), Partner Site Dresden, Germany: German Cancer Research Center (DKFZ), Heidelberg, Germany; Faculty of Medicine and University Hospital Carl Gustav Carus, Technische Universität Dresden, Dresden, Germany, and; Helmholtz Association / Helmholtz-Zentrum Dresden-Rossendorf (HZDR), Dresden, Germany; 6 Department of Nuclear Medicine, Charité – Universitätsmedizin Berlin, corporate member of Freie Universität Berlin, Humboldt-Universität zu Berlin, and Berlin Institute of Health, Berlin, Germany; 7 Department of Radiation Oncology, Charité – Universitätsmedizin Berlin, corporate member of Freie Universität Berlin, Humboldt-Universität zu Berlin, and Berlin Institute of Health, Berlin, Germany; 8 Berlin Institute of Health, Berlin, Germany; 9 Division of Nuclear Medicine, Department of Radiology and Nuclear Medicine, University Hospital Magdeburg, Otto-von-Guericke-Universität Magdeburg, Magdeburg, Germany; 10 Department of Nuclear Medicine, University Hospital Carl Gustav Carus, Technische Universität Dresden, Dresden, Germany

**Keywords:** FDG-PET, Standardized uptake value, SUV, Standardized uptake ratio, SUR, Convolutional neural network

## Abstract

**Purpose:**

The standardized uptake value (SUV) is widely used for quantitative evaluation in oncological FDG-PET but has well-known shortcomings as a measure of the tumor’s glucose consumption. The standard uptake ratio (SUR) of tumor SUV and arterial blood SUV (BSUV) possesses an increased prognostic value but requires image-based BSUV determination, typically in the aortic lumen. However, accurate manual ROI delineation requires care and imposes an additional workload, which makes the SUR approach less attractive for clinical routine. The goal of the present work was the development of a fully automated method for BSUV determination in whole-body PET/CT.

**Methods:**

Automatic delineation of the aortic lumen was performed with a convolutional neural network (CNN), using the U-Net architecture. A total of 946 FDG PET/CT scans from several sites were used for network training (*N* = 366) and testing (*N* = 580). For all scans, the aortic lumen was manually delineated, avoiding areas affected by motion-induced attenuation artifacts or potential spillover from adjacent FDG-avid regions. Performance of the network was assessed using the fractional deviations of automatically and manually derived BSUVs in the test data.

**Results:**

The trained U-Net yields BSUVs in close agreement with those obtained from manual delineation. Comparison of manually and automatically derived BSUVs shows excellent concordance: the mean relative BSUV difference was (mean ± SD) = (– 0.5 ± 2.2)% with a 95% confidence interval of [− 5.1,3.8]*%* and a total range of [− 10.0, 12.0]*%*. For four test cases, the derived ROIs were unusable (< 1 ml).

**Conclusion:**

CNNs are capable of performing robust automatic image-based BSUV determination. Integrating automatic BSUV derivation into PET data processing workflows will significantly facilitate SUR computation without increasing the workload in the clinical setting.

**Electronic supplementary material:**

The online version of this article (10.1007/s00259-020-04991-9) contains supplementary material, which is available to authorized users.

## Introduction

The standardized uptake value (SUV) is currently still the de facto standard for quantitative evaluation in clinical oncological FDG-PET and assumed to be a reasonable surrogate for the metabolic rate of FDG and, ultimately, for tumor glucose consumption. However, the SUV falls short of closely reflecting the latter quantities due to a number of well-known shortcomings. Among these are a notable uptake time dependence, interstudy variability of the arterial input function, and susceptibility to scanner calibration errors [1–3]. Recently, it was shown that the uptake time normalized tumor to blood SUV ratio (standardized uptake ratio, SUR) essentially removes most of these shortcomings which leads to a distinctly improved correlation of this modified uptake measure with the metabolic uptake rate [4–6]. This in turn leads to improved test-retest stability [7] and significantly better prognostic value compared with tumor SUV [8–11].

From a clinical perspective, however, widespread use of SUR is hampered by the fact that its calculation requires knowledge of the image-based blood SUV (BSUV). So far, this value is typically derived from a region-of-interest (ROI) located within the aortic lumen that is manually delineated in the CT image volume of the given PET/CT data while observing crucial constraints such as the necessity to avoid the vicinity of high tracer uptake areas that could induce spillover into the aorta ROI when finally evaluated in the PET data for BSUV determination. This manual ROI delineation requires care and time, thus imposing an additional workload on the clinician. So far, this makes the SUR approach less attractive for clinical routine than the easier to use SUV approach. This situation will only change if the BSUV determination can be automated. We have addressed this issue in the present work.

The task at hand is obviously related to (but distinct from) the similar task of automatic delineation of the whole thoracic aorta in non-contrast-enhanced CT which is a topic that is covered quite extensively in the literature. Traditional approaches to the latter problem exploit the roundness of the aortic cross-section and employ the Hough transform to detect circular shapes in the images [12, 13], utilize labeled multi-atlases [14], or rely on pre-computed anatomy label maps to assist cylinder tracking of the aorta [15]. The major drawback of these methods is their heavy reliance on the used assumptions about aorta position and shape. Therefore, any deviation from these assumptions caused by anatomical abnormalities, implants, or image artifacts can compromise the quality of the results.

Another approach to the aorta delineation problem takes advantage of recent developments in deep learning methods for medical image segmentation. Utilization of convolutional neural networks (CNN) of various architectures—such as 2D and 3D fully convolutional networks [16, 17] and U-Net [18]—leads to state-of-the-art results for the given task. Hybrid approaches have also been described, e.g., the CNN-guided Hough transform-based delineation algorithm [19]. Finally, it was shown [18] that utilization of multiple modalities (e.g., CT and MRI) for fused image segmentation might improve training speed and prediction accuracy.

It is important to realize that automated BSUV determination is not solvable by resorting to existing solutions for CT-only whole-aorta delineation. Specific complications are caused by the inferior spatial and temporal resolution of PET in comparison with CT which can cause substantial partial volume and spillover effects as well as motion artifacts. This would invalidate BSUVs derived from full aorta delineations obtained with established techniques. For these reasons, a dedicated algorithm is necessary, taking into account the full PET/CT data set.

There are three specific constraints that have to be observed during delineation (either manually or algorithmically). First, the delineation needs to be restricted to the central region of the aortic lumen in order to rule out partial volume effects (spill out) that would reduce the measured BSUV. Second, the vicinity of highly FDG-avid regions (such as tumor lesions) must be excluded from the delineation in order to prevent any (even fractionally small) spill in from those regions. Third, the delineation has to avoid areas affected by breathing-induced motion and mismatch between PET and CT that easily can cause attenuation artifacts in abdominal regions.

In this work, we present results obtained with a CNN trained on combined PET/CT data according to the abovementioned requirements and used for automated BSUV determination in a test cohort.

## Methods

### Patients and data acquisition

Altogether, 946 whole-body ^18^F-FDG PET/CTs of 685 patients (445 men, 165 women, 75 unspecified, age 64 ± 8 years) with different tumor diseases (323 lung cancer, 374 esophageal cancer, 249 other) were included. Data acquisition started 76 ± 35 min after injection of 305 ± 66 MBq ^18^F-FDG. 781 out of these 946 scans were contributed by the four clinical sites collaborating in the present study (Germany: Berlin (*N*= 156), Dresden (*N*= 216), Magdeburg (*N*= 119); China: Xiamen (*N*= 290)) using their respective PET/CT systems: Biograph 16 and Biograph mCT 64 (Siemens Medical Solutions, Knoxville, TN, USA), Gemini-TF (Philips Healthcare, Best, The Netherlands), and Discovery STE (GE Medical Systems, Milwaukee, WI, USA).

In addition, data from two prospective multicenter trials conducted by the American College of Radiology Imaging Network (ACRIN 6678, now the ECOG-ACRIN Cancer Research Group, *N*= 67) and by Merck & Co Inc. (MK- 0646-008, shared with ACRIN, *N*= 80) were included. Details on patient groups and PET imaging in these trials can be found in [20]. Furthermore, 18 patients from four Canadian institutions available on the cancer imaging archive (https://wiki.cancerimagingarchive.net) were included [21, 22].

### Ground truth definition

For all 946 datasets, manual delineation of ROIs for BSUV determination within the aortic lumen was performed by an experienced observer (F.H.) who participated in a recent study addressing the interobserver variability of manual BSUV determination [23]. Compared with the latter investigation, delineation was extended to the ascending thoracic aorta as well, otherwise the delineation strategy was identical:

- Both the ascending and descending thoracic aorta were delineated.
- A distance of approximately 8 mm between ROI boundary and aortic wall was required.
- Transaxial planes containing highly FDG-avid structures in close vicinity to the aorta were excluded.
- Aortic arch and abdominal aorta were excluded to avoid motion-induced artifacts.

This strategy ensures avoidance of potentially serious bias in derived BSUV values due to partial volume effects (spill out of aorta signal as well as spill in from the neighbourhood) and motion-induced attenuation artifacts. See the Supplementary Materials for examples of how these effects would otherwise compromise accurate BSUV determination.

### Data preprocessing, network architecture, and training procedure

**Fig. 1:**
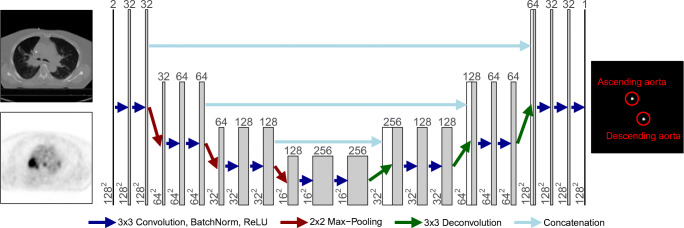
Architecture of the utilized CNN. Numbers above and beside each block designate number of feature channels and matrix size at the given state, respectively. The images on the left are exemplary CT (top) and PET (bottom) images (input) and the image on the right is the corresponding output image (probability map of aorta lumen membership)

The image data processed by the convolutional neural network were prepared as follows. First, PET and CT were resampled to a common voxel grid. For transaxial resampling, the transaxial (x/y) voxel dimensions were set to 2.73 × 2.73 mm in order to reduce the image size (and computational burden during training) while maintaining sufficient spatial sampling and object coverage for the task at hand even after subsequent cropping. The axial (z) voxel dimension was defined by the respective CT data and the corresponding PET data where axially resampled accordingly. After resampling, the image data were transaxially cropped to the central 128 × 128 voxels (corresponding to a transaxial field of view of approximately 35 × 35 cm) in order to further reduce computational burden, especially during training. Finally, all transaxial images were separately normalized such that voxel intensities remain within the [0,1] range. An example of typical resulting PET, CT, and label images is shown in Fig. 1.

The available 946 datasets were split into non-overlapping training (*N*= 293), validation (*N*= 73), and evaluation subsets (*N*= 580). The training subset was used for optimization of the network model parameters while the validation subset was used for monitoring the training process and selection of the best performing model. The evaluation subset was used for assessing the trained network’s performance. Notably, the ACRIN 6678 and Canadian datasets were completely included in the evaluation subset (i.e., completely excluded from the training data). These data were used to investigate potential performance differences when the trained network is applied to data with characteristics potentially different from those used in the training process.

In this work, we employed a modified U-Net architecture [24] as shown in Fig. 1. The network consists of encoder and decoder paths and skip connections which transfer the feature maps from the encoder to the decoder (copy and concatenate). In our implementation, we use 3 × 3 zero-padded convolutions followed by batch-normalization and ReLU activation layers. A 2 × 2 max-pooling with stride 2 was used for downsampling and 3 × 3 deconvolution was employed for upsampling. Each downsampling operation is accompanied by a factor of two increase in the feature channel number while upsampling decreases this number by the same factor. We found it sufficient to use 32 output feature channels in the first convolution and to perform a total of 3 downsampling and upsampling operations, respectively.

The network was implemented and trained with the Apache MXNet (version 1.5.1) package for the R language and environment for statistical computing (version 3.6.3). The training was performed with RMSprop optimizer (learning rate = 0.001, mini-batch size = 64) for 250 epochs. The logistic loss function was optimized in the training. The training process was monitored with a dedicated metric designed to estimate the mean plane-wise BSUV error as follows. For each PET image *i*, the averages $\text {BSUV}_{\text {man}}^{i}$ and $\text {BSUV}_{\text {CNN}}^{i}$ over manual and CNN ROI delineation are computed. For images where either of these two delineations is missing (unlabeled in the test data or not delineated by network), the respective mean BSUV in the given batch is used instead (if both delineations are missing the respective image is skipped). Finally, the absolute relative BSUV differences, $\left |(\text {BSUV}_{\text {CNN}}^{i} - \text {BSUV}_{\text {man}}^{i}) \big / \text {BSUV}_{\text {man}}^{i}\right |$, are averaged within and over the batches to yield the final score. The model which achieved the lowest score on the validation dataset was considered the optimally trained network. In order to prevent fast overfitting, data augmentation in the form of non-rigid warp transforms was applied during the training.

The values of the hyperparameters were selected among other possible choices as offering the highest BSUV concordance and smallest number of failed delineations (yielding ROI volumes < 1 ml). Also, the chosen U-Net architecture outperformed the dilated CNN [25, 26]. Especially, the latter architecture caused larger BSUV deviations in test data from sites not contributing to the training dataset and was therefore discarded.

### Model evaluation

For each study in the evaluation dataset, a probability map for ROI membership of the individual voxels was generated with the trained network model. ROIs were derived from the probability maps by applying a threshold of 0.5. If the resulting ROI volume was < 1 ml, the delineation was considered unusable. For the remaining ROIs (and the corresponding manual delineations), BSUVs were determined as ROI averages using the ROVER software (version 3.0.51; ABX GmbH, Radeberg, Germany). We quantify the relative difference of both BSUV predictions according to:

1 $$ \Updelta \text{BSUV} = \frac{\text{BSUV}_{\text{CNN}} - \text{BSUV}_{\text{man}}}{\text{BSUV}_{\text{man}}}, $$

where BSUV_man_ and BSUV_CNN_ are manually and automatically derived BSUV values, respectively.

## Results

Training of the network was performed on a dual-socket CPU computational node (2 × 14 cores Intel Xeon E5-2690 v4, 392 GB RAM). Total training time using our training (*N*= 60190 image planes) and validation (*N*= 14525 image planes) PET/CT datasets amounted to about 80 h for the finally selected network architecture. Processing time for a single PET/CT dataset using the trained network amounted to about 4 s on 8 CPU cores.

**Fig. 2:**
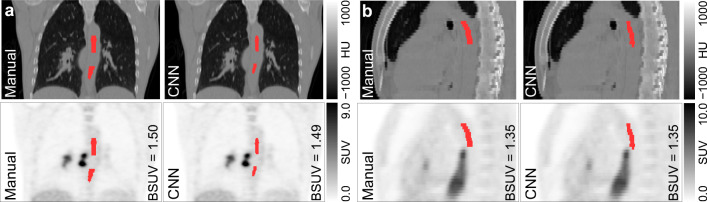
Comparison of manual and CNN-based aortic lumen delineation for two patients with **a** lung cancer (coronal view) and **b** esophageal cancer (sagittal view). CT (top) and corresponding PET (bottom) images are overlaid by the respective delineations (shown in red). Note that the CNN excludes the vicinity of the lesions from delineation in a way similar to the human observer

Figure 2 provides an exemplary visual comparison of the automatically and manually delineated ROIs for two typical test cases of patients suffering, respectively, from esophageal and lung cancers. In both cases, malignant lesions exhibiting high FDG uptake in the PET images are located adjacent to the aortic wall. As can be seen, the trained network is capable of mimicking rather closely the decision of the human observer to exclude these areas as well as the potentially breathing motion-affected region near and below the diaphragm from the aorta lumen ROI. Consequently, the manually and automatically derived BSUVs are virtually identical in these two examples.

**Fig. 3:**
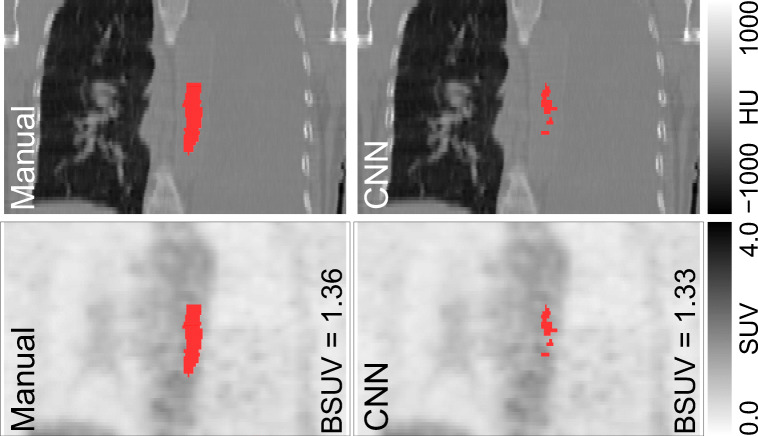
Comparison of manual and CNN-based aortic lumen delineation for a patient with pleural effusion, atelectasis of the left lung and ectasia of the ascending aorta. Coronal CT (top) and corresponding PET (bottom) images are overlaid by the respective delineations (shown in red). Note that the CNN struggles to fully identify the aortic lumen for this untypical test case, resulting in a distinctly smaller volume of the resulting ROI than is the case with manual delineation

Figure 3 shows an untypical test case (from a patient with pleural effusion and atelectasis in the left lung and ectasia of the ascending aorta) that is not adequately represented in the training data. As can be seen, the CT image is heavily altered, accordingly. While the human observer can abstract from these confounding image characteristics and define a ROI similar to other, more typical test cases, the network yields a distinctly smaller number of voxels in the generated probability map that exceed the chosen 0.5 threshold. Consequently, the resulting ROI has only a rather small volume of 1.65 ml near the chosen limit of 1 ml below which delineation would have been considered a failure. Despite the significant deviation of the network’s ROI from the manual delineation in this case, the resulting BSUV difference remains modest (Δ*BSUV* = − 2.6*%*) reflecting the fact that both ROIs still can be considered acceptable choices regarding the BSUV determination task.

**Fig. 4:**
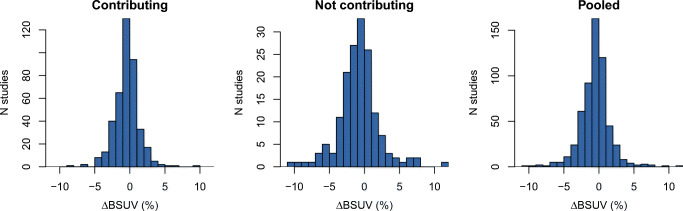
ΔBSUV histograms for the evaluation datasets. Results for sites selected to contribute to the training dataset (left) are shown separately from those which were not (middle). Pooled results (right) represent the data from all participating sites

**Fig. 5:**
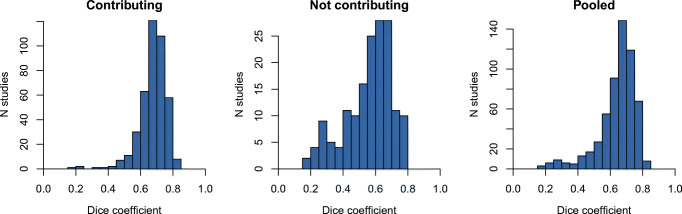
Dice coefficient histograms for the evaluation datasets. Results for sites selected to contribute to the training dataset (left) are shown separately from those which were not (middle). Pooled results (right) represent the data from all participating sites

Considering the pooled results for all test cases, the observed frequency distribution of relative differences between manual and automatic BSUV determination is shown in Fig. 4. Corresponding summary statistics are provided in Table 1 and the related frequency distribution of Dice coefficients is shown in Fig. 5. As can be seen, differences between manual and automatic BSUV never exceeded 15% and the 95% confidence interval of observed differences stays within (0 ± 7)%. There is a notable tendency for somewhat larger deviations for test cases from sites that were completely excluded from contributing to the training and validation data: all cases exceeding 10% deviation are found in this subset. Nevertheless, the effect size is only modest.

**Table 1:** Evaluation results. The results for data from sites included and sites not included in the training set are shown separately

Site contributing to training data	Sample size	Failed (< 1 ml)	ΔBSUV (%)		
			Mean ± SD	95*%* CI	Range
Yes	415	2	− 0.4 ± 1.7	[− 4.1, 2.8]	[− 8.3, 9.7]
No	165	2	− 0.8 ± 3.0	[− 6.7, 6.1]	[− 10.0, 12.0]
Pooled	580	4	− 0.5 ± 2.2	[− 5.1, 3.8]	[− 10.0, 12.0]

## Discussion

The present proof-of-concept study demonstrates that a suitable convolutional neural network, trained on combined PET/CT data, does allow fully automated image-based BSUV determination with a performance that is overall comparable with that of a typical experienced human observer. This assessment follows from the fact that the trained network achieves concordance with the given human observer (that also defined the ground truth delineation used for network training) at a level that is comparable with the concordance achieved between different human observers.

It is important to realize in this context that the delineation’s objective, as outlined in the Methods section, is somewhat less precisely defined than e.g. delineation of the aortic wall or the full aortic lumen since the focus is on accurate and reproducible BSUV determination rather than on exact delineation of a well-defined anatomical structure. Moreover, the criterion used for optimal network selection is chosen in such a way as to optimize concordance of derived BSUVs rather than to achieve optimal geometrical concordance of the resulting ROI delineations. Both factors combined explain the modest Dice coefficients observed in the present study (around 0.6–0.7, see Fig. 5) which are distinctly smaller than what is achievable with networks optimized for classical organ segmentation tasks. This is also in accord with the observation that there is only a rather weak negative correlation (*r*=− 0.45) between achieved Dice coefficient and |ΔBSUV|, demonstrating the intuitively obvious fact that similar BSUV values can be obtained from somewhat differing ROI delineations within the given aortic lumen (see Fig. 3).

The residual delineation ambiguities inherent in the given task are also reflected in the results of a recent study comparing interobserver variability of manual aorta lumen delineation and BSUV determination: the results shown in Table 2 of [23] might be compared with the results of the present investigation as summarized in Table 1. In the former work, Δ*BSUV*_*dp*_ represents the interobserver variability of BSUV determination within a group of 8 experienced observers using the observer-averaged BSUV for each patient as the ground truth. The results reported there (SD = 2.77%, 95%CI = [− 5.87,5.50]%, range = [− 10.6,10.8]%) rather closely compare with the corresponding pooled results in Table 1.

This situation might be paraphrased in this way: for the given task, the interobserver variability of image-based BSUV determination in a group of experienced human observers is very similar to the deviations observed in the present study between the automated observer (the trained CNN) and the human observer which defined the ground truth used for training of the CNN (and which also took part in the mentioned interobserver study). In this sense, it can be stated that the automated observer performs comparable with a representative experienced human observer.

This statement holds true even if closer inspection of Table 1 demonstrates that agreement between CNN and human observer is somewhat reduced in the subset of test data from sites that did not contribute any training data (which thus are different regarding details of imaging protocols, image quality, and spatial resolution of both PET and CT). In comparison with the other subset (test data from sites also contributing training data), standard deviation of ΔBSUV increases from 1.7 to 3.0% and the 95% confidence interval from about ± 4 to ± 7%. Nevertheless, the level of agreement between CNN and human observer is completely sufficient in both subsets keeping in mind that the ultimate objective of BSUV determination is its use in SUR computation in order to account for inter-patient BSUV variability which is characterized by a typical standard deviation of about 16% [9, 23, 27]. It is also worth to re-emphasize that the spurious patient-dependent BSUV bias which would be introduced by a solely CT-based anatomical whole-aorta delineation (be it algorithmically or manually) would be of the same order of magnitude as the actual inter-patient BSUV variability and, therefore, such an approach would be completely unsuitable for the task at hand (also see the examples provided in Supplementary Materials).

Regarding usefulness for actual application in a clinical research setting (or even in clinical routine), it is especially important that the trained network is able to reliably mimic the human observer regarding omission of certain areas from the delineation that could otherwise lead to serious errors in BSUV determination. Notably, the good performance of our model is tightly related to its ability to exclude the vicinity of FDG-avid regions from the delineation as illustrated in Fig. 2. Depending on considered tumor entity, such cases can make up a sizable fraction of all investigated patients. The ability to handle these cases correctly is achieved by utilizing combined PET/CT data rather than CT data alone for training despite the fact that the anatomical information regarding location of the aortic lumen is mostly provided by the CT data. Indeed, while training based on the CT data alone—keeping the objective to exclude regions excluded by the human observer based on consideration of the PET data—is possible (since many FDG-avid tumors also are identifiable in the CT data), the resulting networks perform inferior (data not shown) to the one trained on the combined PET/CT images: the PET information clearly is not redundant as far as optimal training of the network is concerned.

Manifest failure of the automatic BSUV determination (signaled by ROI volumes below 1 ml) was observed in only 4 out of 580 test datasets. A closer look at these 4 cases reveals that all of them are associated with significant anatomic abnormalities affecting size, shape, and appearance of one or both lungs and/or location and shape of the aorta. Reliable handling of such unusual cases by the network would require their adequate representation in the training data which, given their rare occurrence, would require to increase the total size of the training data set substantially in order to represent the extreme margins of anatomical variability in sufficiently large numbers. However, a failure rate of < 0.7% (and the concomitant rare necessity for human intervention and handling of these cases) seems already quite acceptable for use of such a network for BSUV determination in a clinical context.

The current work has some obvious limitations. First of all, the number of studies (*N*= 293) and, consequently, the number of separate 2D image slices used for network training (*N*= 60190) are comparatively small considering the typical demands of deep learning applications. This is a common problem of utilizing deep learning techniques for delineation tasks in tomographic medical imaging, since manual labeling of volumetric image data is a very time-consuming process. However, as many previous investigations were able to demonstrate, it is possible to achieve impressive results with limited numbers of training datasets if proper approaches to diminish the data scarcity effects are used [28, 29]. In our work, we employed data augmentation via non-rigid image deformations and over-fitting control using the validation dataset to minimize the consequences of limited training data. Nevertheless, it definitely would be desirable to successively augment the training dataset and to retrain the network. This might especially help to further reduce the fraction of failed delineations to negligible levels and also to reduce the magnitude of the maximally observed deviations between network and human observer which currently still are somewhat larger (although not critically so) than those between two human observers.

Another principal issue is the quality of the ground truth, i.e., the aorta lumen delineations provided in the training data. These were performed by a single experienced observer which introduces a certain level of subjectivity. The modest extent of this subjectivity and case-to-case variability is characterized in the mentioned interobserver study [23]. It would nevertheless have been desirable to train the network based on delineations performed by multiple observers but this was not doable considering the given time resources for the present study.

The presented network model, therefore, can not be expected to provide objectively optimal results (which in the present context would mean to act as the average of many experienced human observers). Rather, the model is trained to mimic the single human observer that defined the training data delineations and served as reference in the evaluation data as well. In view of the actually quite small interobserver variability demonstrated in [23] this is, however, not a relevant shortcoming in our opinion. Nevertheless, our approach will have to prove its viability in dedicated future studies to demonstrate that superior prognostic value of SUR in comparison with SUV is maintained even if SUR computation is utilizing automated BSUV determination as proposed in the present work.

## Conclusion

CNNs are capable of performing robust automatic image-based BSUV determination. The U-Net model used in this work performs comparable with an experienced human observer. Integrating automatic BSUV determination into PET data processing workflows will significantly facilitate SUR computation and might allow its use as a superior drop-in replacement for SUV-based quantitation without increasing the workload in the clinical setting.

## Electronic supplementary material

Below is the link to the electronic supplementary material.

(PDF 230 KB)

## References

[1] Hamberg L, Hunter G, Alpert N, Choi N, Babich J, Fischman A (1994). The dose uptake ratio as an index of glucose metabolism: useful parameter or oversimplification?. J Nucl Med.

[2] Keyes JJr (1995). SUV: Standard Uptake or silly useless value?. J Nucl Med.

[3] Huang S (2000). Anatomy of SUV. Nucl Med Biol.

[4] Van den Hoff J, Oehme L, Schramm G, Maus J, Lougovski A, Petr J (2013). The PET-derived tumor-to-blood standard uptake ratio (SUR) is superior to tumor SUV as a surrogate parameter of the metabolic rate of FDG. EJNMMI Res.

[5] Van den Hoff J, Lougovski A, Schramm G, Maus J, Oehme L, Petr J (2014). Correction of scan time dependence of standard uptake values in oncological PET. EJNMMI Res.

[6] Hofheinz F, Van den Hoff J, Steffen IG, Lougovski A, Ego K, Amthauer H (2016). Comparative evaluation of SUV, tumor-to-blood standard uptake ratio (SUR), and dual time point measurements for assessment of the metabolic uptake rate in FDG PET. EJNMMI Res.

[7] Hofheinz F, Apostolova I, Oehme L, Kotzerke J, Van den Hoff J (2017). Test–retest variability in lesion suv and lesion SUR in 18F-FDG PET: an analysis of data from two prospective multicenter trials. J Nucl Med.

[8] Bütof R, Hofheinz F, Zöphel K, Stadelmann T, Schmollack J, Jentsch C (2015). Prognostic value of pretherapeutic tumor-to-blood standardized uptake ratio in patients with esophageal carcinoma. J Nucl Med.

[9] Hofheinz F, Bütof R, Apostolova I, Zöphel K, Steffen IG, Amthauer H (2016). An investigation of the relation between tumor-to-liver ratio (TLR) and tumor-to-blood standard uptake ratio (SUR) in oncological FDG PET. EJNMMI Res.

[10] Bütof R, Hofheinz F, Zöphel K, Schmollack J, Jentsch C, Zschaeck S (2019). Prognostic value of SUR in patients with trimodality treatment of locally advanced esophageal carcinoma. J Nucl Med.

[11] Hofheinz F, Li Y, Steffen IG, Lin Q, Lili C, Hua W (2019). Confirmation of the prognostic value of pretherapeutic tumor SUR and MTV in patients with esophageal squamous cell carcinoma. Eur J Nucl Med Mol Imaging.

[12] Kurkure U, Avila-Montes OC, Kakadiaris IA. Automated segmentation of thoracic aorta in non-contrast CT images. 2008 5th IEEE International Symposium on Biomedical Imaging: From Nano to Macro. IEEE; 2008. p. 29–32, 10.1109/ISBI.2008.4540924.

[13] Kurugol S, San Jose Estepar R, Ross J, Washko GR. Aorta segmentation with a 3D level set approach and quantification of aortic calcifications in non-contrast chest CT. 2012 Annual International Conference of the IEEE Engineering in Medicine and Biology Society. IEEE; 2012. p. 2343–2346, 10.1109/EMBC.2012.6346433. 10.1109/EMBC.2012.6346433 PMC3671590 23366394

[14] Išgum I, Staring M, Rutten A, Prokop M, Viergever M, Van Ginneken B (2009). Multi-atlas-based segmentation with local decision fusion–application to cardiac and aortic segmentation in CT scans. IEEE Trans Med Imaging.

[15] Xie Y, Padgett J, Biancardi AM (2014). Reeves AP. Automated aorta segmentation in low-dose chest CT images. Int J Comput Assist Radiol Surg.

[16] Noothout J, De Vos B, Wolterink J, Išgum I. Automatic segmentation of thoracic aorta segments in low-dose chest CT. Medical Imaging 2018: Image Processing. In: Angelini ED and Landman BA, editors; 2018. International Society for Optics and Photonics, SPIE, 105741S. 10.1117/12.2293114.

[17] Van Harten LD, Noothout JMH, Verhoeff JJC, Wolterink JM, Išgum I. Automatic segmentation of organs at risk in Thoracic CT scans by combining 2D and 3D convolutional neural networks. SegTHOR challenge, ISBI; 2019. p. 1–4.

[18] Wang D, Zhang R, Zhu J, Teng Z, Huang Y, Spiga F, et al. Neural network fusion: a novel CT-MR Aortic Aneurysm image segmentation method. Medical Imaging 2018: Image Processing. In: Angelini ED and Landman BA, editors; 2018. International Society for Optics and Photonics, SPIE, 1057424. 10.1117/12.2293371. 10.1117/12.2293371 PMC6067661 30072821

[19] Mohammadi S, Mohammadi M, Dehlaghi V, Ahmadi A (2019). Automatic segmentation, detection, and diagnosis of abdominal aortic aneurysm (AAA) using convolutional neural networks and hough circles algorithm. Cardiovasc Eng Technol.

[20] Weber WA, Gatsonis CA, Mozley PD, Hanna LG, Shields AF, Aberle DR (2015). Repeatability of 18F-FDG PET/CT in advanced non–small cell lung cancer: prospective assessment in 2 multicenter trials. J Nucl Med.

[21] Clark K, Vendt B, Smith K, Freymann J, Kirby J, Koppel P (2013). The cancer imaging archive (TCIA): maintaining and operating a public information repository. J Digit Imaging.

[22] Vallieres M, Kay-Rivest E, Perrin LJ, Liem X, Furstoss C, Khaouam N, et al. 2017. Data from head-neck-PET-CT The Cancer Imaging Archive.

[23] Hofheinz F, Maus J, Zschaeck S, Rogasch J, Schramm G, Oehme L (2019). Interobserver variability of image-derived arterial blood SUV in whole-body FDG PET. EJNMMI Res.

[24] Ronneberger O, Fischer P, Brox T. U-Net: Convolutional Networks for Biomedical Image Segmentation. Medical Image Computing and Computer-Assisted Intervention – MICCAI. In: Navab N, Hornegger J, Wells WM, and Frangi AF, editors. Cham: Springer International Publishing; 2015. p. 234–241, 10.1007/978-3-319-24574-4_28.

[25] Wolterink JM, Leiner T, Viergever MA, Išgum I. Dilated convolutional neural networks for cardiovascular MR segmentation in congenital heart disease. Reconstruction, Segmentation, and Analysis of Medical Images. In: Zuluaga MA, Bhatia K, Kainz B, Moghari MH, and Pace DF, editors. Cham: Springer International Publishing; 2017. p. 95–102, 10.1007/978-3-319-52280-7_9.

[26] Yu F, Koltun V. Multi-scale context aggregation by dilated convolutions. 4th international conference on learning representations, ICLR, May; 2016. p. 1–13.

[27] Boktor RR, Walker G, Stacey R, Gledhill S, Pitman AG (2013). Reference range for intrapatient variability in blood-pool and liver SUV for 18F-FDG PET. J Nucl Med.

[28] Hesamian MH, Jia W, He X, Kennedy P (2019). Deep learning techniques for medical image segmentation: achievements and challenges. J Digital Imaging.

[29] Tajbakhsh N, Jeyaseelan L, Li Q, Chiang JN, Wu Z, Ding X (2020). Embracing imperfect datasets: a review of deep learning solutions for medical image segmentation. Med Image Anal.

