# Supplementary material for: A convolutional neural network for fully automated blood SUV determination to facilitate SUR computation in oncological FDG-PET
Source: Eur J Nucl Med Mol Imaging. 2020 Oct 1;48(4):995–1004. doi: 10.1007/s00259-020-04991-9 (PMC8041711; doi:10.1007/s00259-020-04991-9)
Supplement: Supplementary file 1 — (PDF 230 KB) [file 259_2020_4991_MOESM1_ESM.pdf]

European Journal of Nuclear Medicine and Molecular Imaging

Supplementary material:

A convolutional neural network for fully automated blood SUV determination in oncological FDG-PET

Pavel Nikulin [1,*], Frank Hofheinz [1], Jens Maus [1], Yimin Li [2], Rebecca Bütof [3,4,5], Catharina Lange [6], Christian Furth [6], Sebastian Zschaeck [7,8], Michael C. Kreissl [9], Jörg Kotzerke [10], Jörg van den Hoff [1,10]

[1]*Helmholtz-Zentrum Dresden-Rossendorf, PET Center, Institute of Radiopharmaceutical Cancer Research*
[2]*Department of Radiation Oncology, Xiamen Cancer Center, The First Affiliated Hospital of Xiamen University, Xiamen, China*
[3]*OncoRay – National Center for Radiation Research in Oncology, Faculty of Medicine and University Hospital Carl Gustav Carus, Technische Universität Dresden, Helmholtz-Zentrum Dresden-Rossendorf, Dresden, Germany*
[4]*Department of Radiotherapy and Radiation Oncology, Faculty of Medicine and University Hospital Carl Gustav Carus, Technische Universität Dresden, Dresden, Germany*
[5]*National Center for Tumor Diseases (NCT), Partner Site Dresden, Germany: German Cancer Research Center (DKFZ), Heidelberg, Germany; Faculty of Medicine and University Hospital Carl Gustav Carus, Technische Universität Dresden, Dresden, Germany, and; Helmholtz Association / Helmholtz-Zentrum Dresden-Rossendorf (HZDR), Dresden, Germany*
[6]*Department of Nuclear Medicine, Charité – Universitätsmedizin Berlin, corporate member of Freie Universität Berlin, Humboldt-Universität zu Berlin, and Berlin Institute of Health, Berlin, Germany*
[7]*Department of Radiation Oncology, Charité – Universitätsmedizin Berlin, corporate member of Freie Universität Berlin, Humboldt-Universität zu Berlin, and Berlin Institute of Health, Berlin, Germany*
[8]*Berlin Institute of Health, Berlin, Germany*
[9]*Division of Nuclear Medicine, Department of Radiology and Nuclear Medicine, University Hospital Magdeburg, Otto-von-Guericke-Universität Magdeburg, Magdeburg, Germany*
[10]*Department of Nuclear Medicine, University Hospital Carl Gustav Carus, Technische Universität Dresden, Dresden, Germany*

This document is intended to illustrate with exemplary patient data the necessity of a PET/CT based strategy for BSUV determination as detailed in the present paper rather than resorting to existing approaches for CT based anatomical delineation of the whole aorta.

The line plots in the Figure below show a comparison of manual aorta delineation with the approach described in the "Ground truth definition" paragraph of the Methods section using the combined PET/CT information (red profiles) and manual whole-aorta delineation based solely on the CT images (blue profiles). Results for three different patients are shown. The delineations were performed in the ascending aorta as well as in the descending aorta from aortic arch to abdomen (when applicable) using the transaxial slices. The line plots in the top row show the slice based ROI averages (SUV units) for the included transaxial slices. Middle and bottom row show representative sagittal slices of the underlying CT and PET data, respectively. The result of the manual whole-aorta delineation is indicated by the blue ROI outline.
Three exemplary cases are shown to separately illustrate the major potential pitfalls of using a CT-only whole-aorta delineation for BSUV determination.

1. Left column: this example demonstrates the partial volume effect (spill out from aorta) when delineating the full aortic lumen without observing the 8 mm safety margin between wall and ROI boundary in the transaxial slices. The effect size in this example is 8% and can be calculated to usually fall into the 10-20% range in case of a perfectly cold background.

*Bautzner Landstrasse 400, 01328 Dresden, Germany. E-mail: p.nikulin@hzdr.de

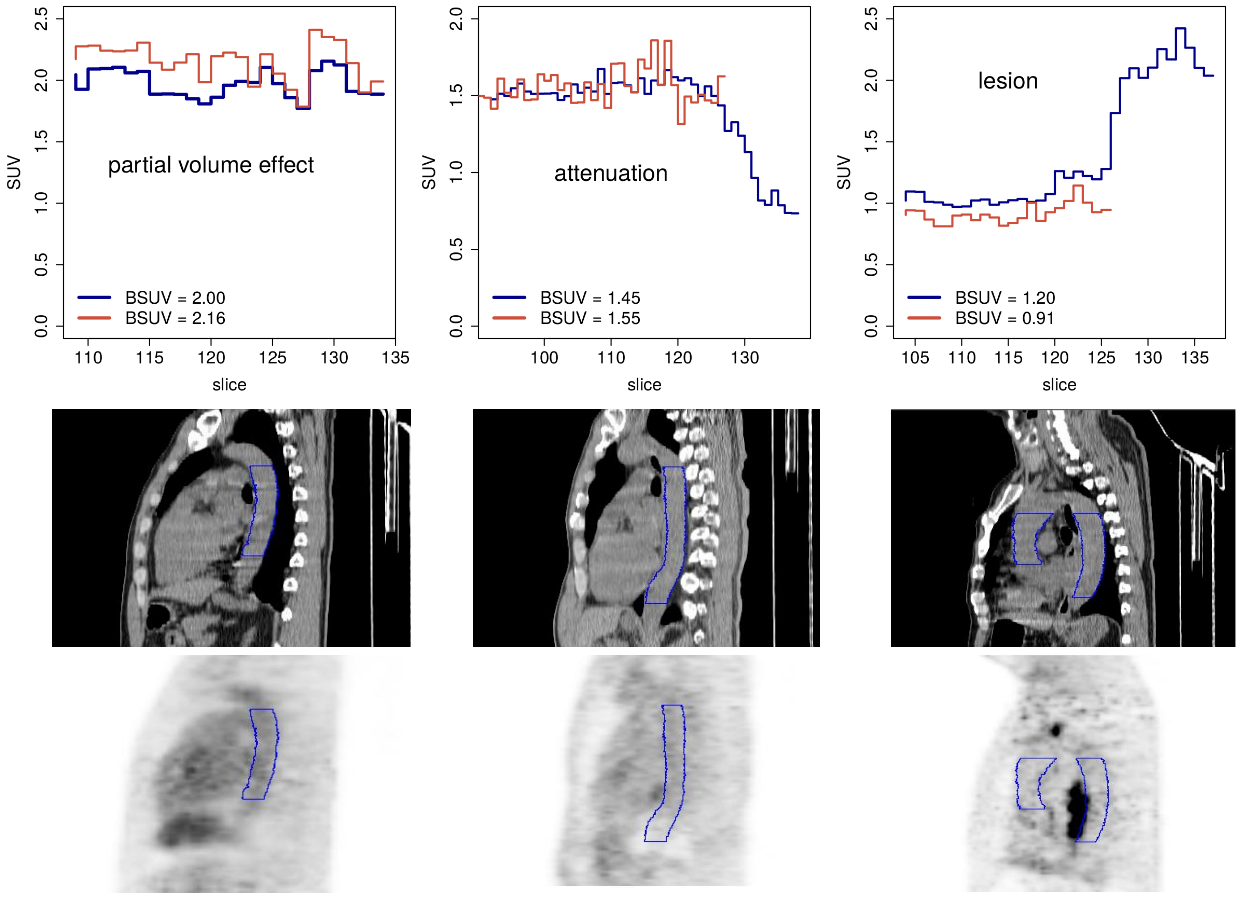

2. Middle column: this example illustrates the signal drop caused by motion-induced attenuation artifacts (PET CT mismatch near the abdomen). Erroneously including the affected slices causes a 7% bias in this example and might become distinctly larger in more extreme cases.

3. Right column: this example illustrates the influence of spill in into the aorta ROI from a neighboring FDG-avid lesion. Note the additional presence of visible modest tracer accumulation in the aortic wall leading to further increase of spill in into the whole aorta ROI. The latter is essentially eliminated by use of the 8 mm safety margin while the former requires complete exclusion of the affected slices due to the extremely high signal from the lesion. In the present example, the whole-aorta delineation causes a BSUV bias exceeding 30%.

References

[1] Hofheinz F, Maus J, Zschaeck S, Rogasch J, Schramm G, Oehme L, et al. Interobserver variability of image-derived arterial blood SUV in whole-body FDG PET. EJNMMI Research. 2019;9:23. https://doi.org/10.1186/s13550-019-0486-9.
